# Dual-mobility implants in primary and revision total hip arthroplasty: A systematic review and meta-analysis

**DOI:** 10.1016/j.jcot.2024.102495

**Published:** 2024-07-18

**Authors:** Sarup Saroha, Firas J. Raheman, Parag K. Jaiswal, Akash Patel

**Affiliations:** a University College London, London, United Kingdom; b Royal Free London NHS Foundation Trust, London, United Kingdom

**Keywords:** Dislocation, Dual-mobility implants, Implant failure, Meta-analysis, Post-operative complications, Revision surgery, Systematic review, Total hip arthroplasty, Total joint replacement

## Abstract

**Purpose:**

Total hip arthroplasty (THA) is a common and successful operation. However, dislocation remains a significant cause of implant failure in fixed-bearing designs. This study investigated the effect of dual-mobility implants (DM) compared to fixed-bearing (FB) implants on all-cause revisions, revisions due to dislocation, post-operative complications and functional scores in patients undergoing primary and revision THA.

**Methods:**

A systematic review was performed including studies that compared DM with FB implants in primary or revision THA according to PRISMA guidelines, and was registered in PROSPERO (ID CRD42023403736). The Cochrane Library, Embase, MEDLINE, Web of Science, and Scopus were searched from the time of database inception to March 12, 2023. Eligible studies underwent meta-analysis and risk of bias assessment using the ROBINS-I tool. Treatment effects were assessed using odds ratios and data were pooled using a random-effects maximum-likelihood, where appropriate.

**Results:**

Eight comparative, non-randomised studies involving 2810 DM implants and 3188 FB implants were included. In primary THA, there was an imprecise estimate of the difference in all-cause revision (OR 0.82, 95 % CI 0.25–2.72) and a significant benefit for the DM cohort in revision due to dislocation (OR 0.08, 95 % CI 0.02–0.28). In revision THA, the DM cohort showed benefit in all-cause revision (OR 0.57, 95 % CI 0.31–1.05) and revision due to dislocation (OR 0.14, 95 % CI 0.04–0.53). DM implants were associated with a lower incidence of implant dislocation and infection. The analysis of functional outcomes was limited due to reporting limitations. No intraprosthetic dislocations were observed.

**Conclusion:**

The results suggest that contemporary DM designs may be advantageous in reducing the risk of all-cause revision, revision due to dislocation, and post-operative complication incidence at mid-term follow-up. Further high-quality prospective studies are needed to evaluate the long-term risk profile of this design, especially in the revision context.

## 1 Introduction

Total Hip Arthroplasty (THA) involves replacing a hip joint with an artificial implant to alleviate hip pain and improve patient mobility. The Lancet has hailed it as one of the world's most successful surgical procedures, naming it the “operation of the century”.^1^ The National Joint Registry (NJR) reported 84,998 primary THAs (pTHAs) in 2021, confirming its ubiquity.^2^

However, dislocation remains a significant cause of implant failure after THA.^2^ The NJR reported that the second most common indication for a revision procedure after pTHA was a dislocation or subluxation of an implant.^2^ Furthermore, an epidemiological study conducted in the USA revealed that amongst 51,345 revision THAs (rTHAs) examined, 22.5 % were due to dislocations.^3^

Dislocation after THA significantly impacts a patient's quality of life,^4^ causing pain, discomfort, and limited mobility. It also has significant economic and societal impacts, as consequent closed reductions and revision surgery increase patient healthcare costs by 19 % and 148 %, respectively.^5^

The fixed-bearing (FB) THA model has been developed since its conception in 1962 to enhance hip range of motion (ROM) whilst reducing pain.^6^^,^^7^ It employs a small-diameter prosthesis combined with a high molecular weight polyethylene liner, dampening force transmission.

To decrease the incidence of dislocation, modern designs including large-diameter femoral heads, constrained and elevated-rim acetabular implants have been developed.^8^^,^^9^

Annual primary and revision THA rates are set to increase due to an increasingly active, ageing, and obese population.^10^, ^11^, ^12^, ^13^ Hence, interest in dual-mobility (DM) implant design has been rising.

**Fig. 1:**
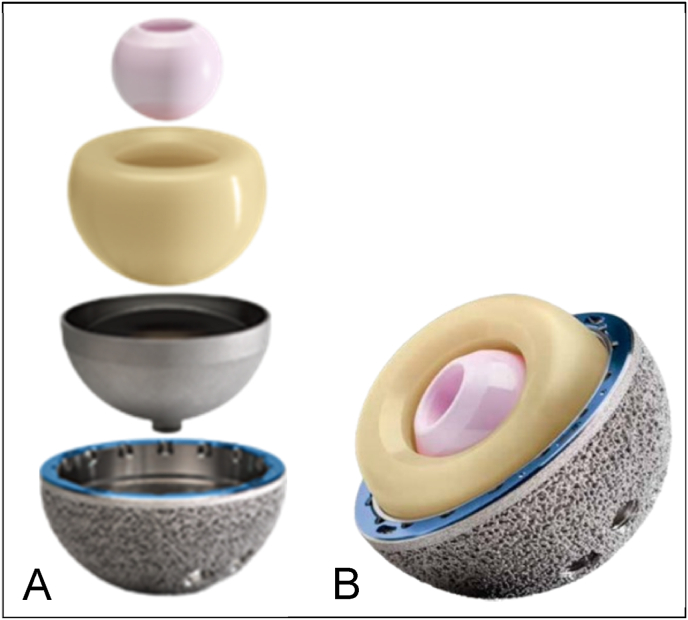
(A) Exploded view of a Zimmer Biomet G7 dual mobility acetabular system (B) Assembled view of a Zimmer Biomet G7 dual mobility acetabular system. The small ceramic head, large liner, outer metal cup, and acetabular shell are arranged from top to bottom and inside to outside in (A) and (B), respectively. The primary articulation occurs between the small ceramic head and the large polyethylene liner (Images courtesy of Zimmer Biomet, Warsaw).

Bousquet and Rambert introduced the DM concept in 1974,^14^ aiming to improve stability and reduce dislocation rates by decreasing impingement and increasing physiologically effective head size.^15^ Charnley's low-friction principle is applied to the small articulation surface between the femoral head and the polyethylene liner, improving prosthesis durability in combination with the McKee-inspired large femoral head between the polyethylene liner and the outer metal cup^6^ (Fig. 1).

The DM design provides a greater ROM and theoretical jump distance that may give better stability and a lower risk of dislocation.^16^^,^^17^ However, the extended sliding distance of the femoral head across the acetabular cup with an additional joint bearing surface may generate increased frictional torque, causing wear, osteolysis, and loosening.^18^

Despite high prosthesis costs, the DM design is cost-effective,^19^, ^20^, ^21^ and it was approved by the USFDA in 2009, leading to an uptake in its use.^22^ Studies have indicated the model's promise in high-risk patient populations,^23^, ^24^, ^25^, ^26^ but there remains a paucity of information regarding its use in the primary and revision settings.

By pooling the available comparative evidence, a better understanding of the efficacy of the DM and FB models could be gained to improve implant selection and improve patient outcomes. This study, therefore, aimed to compare DM implants and FB implants in primary and revision THA settings by the outcomes of all-cause revision, revision due to dislocation, post-operative complications, and clinical scores at the latest follow-up.

## 2 Materials and methods

This systematic review and meta-analysis adhered to Preferred Reporting Items for Systematic Review and Meta-Analysis (PRISMA) guidelines,^27^ and to a pre-defined protocol that was registered *a priori* in PROSPERO (CRD42023403736). The eligibility criteria for this systematic review are provided in Table 1.

**Table 1:** Eligibility criteria.

Domain	Inclusion Criteria	Exclusion Criteria
Population	Adult patients (≥18 years old) of either sex undergoing pTHA or rTHA.	Children (age <18).
		Femoral neck fracture cases.
		Neurologic disease cases.
Intervention	Any acetabular shell design.	Hemiarthroplasties.
	Cemented or uncemented femoral components.	Resurfacing arthroplasties.
	Any surgical approach.	Arthrodesis.
Comparison	FB THA implants.	Studies that did not compare DM implants with FB implants.
Outcome	Studies clearly reporting patient functional outcomes and complications data.	Studies that did not clearly report primary or secondary outcome data.
Study Design	Randomised controlled trials.	Abstracts.
	Quasi-randomised controlled trials.	Anatomical studies.
	Prospective or retrospective comparative studies.	Biomechanical studies.
	Case-controlled studies.	Cadaveric studies.
		Case reports.
		Case series.
		Commentaries.
		Diagnostic studies.
		Duplicate studies.
		Expert opinions.
		Intraoperative validation studies.
		Letters to the editor.
Context	Studies published by peer-reviewed journals.	Studies that did not clearly separate data regarding primary cases from revision cases.
	Any language.	
	Any date of publication.	
	Any duration of follow-up.	

The online databases of CENTRAL; CDSR; Embase; MEDLINE; Web of Science (Science Citation Index); and Scopus were systematically searched for relevant studies. CENTRAL and CDSR were searched via the Cochrane Library. Embase and MEDLINE were searched via Ovid. The search strategies (Supplementary File 1) used a PICO-style approach that combined free text and controlled vocabulary (MeSH) search terms according to the eligibility criteria.

Databases were searched from inception to the time of study retrieval, March 12, 2023. Eligible studies were cross-referenced for additional studies, and the “Cited Reference Search” function in the Web of Science database was used on April 12, 2023 to aid in the complete capture of the literature. Relevant study authors were contacted for clarification if there was uncertainty.

Two authors (SS, FJR) performed independent record title and abstract screening. A third author (AP) arbitrated in the case of any disagreement. Full-text reports were then sought and retrieved for all records to evaluate them against the eligibility criteria.

Search results were imported into Rayyan, a web-based citation manager.^28^ No artificial intelligence or machine-learning algorithms were employed at any stage.

Two authors (SS, FJR) independently extracted data from eligible studies to a piloted database in Microsoft Excel®. Various data were retrieved from eligible studies, including article details, study design, population demographics, and operative details.

The primary outcome for this study was all-cause surgical revision at the latest follow-up. As per the NJR 19th Annual Report 2022,^2^ a revision was defined as “any operation where one or more components are added to, removed from or modified in a joint replacement”.

The secondary outcome was revision due to dislocation at the latest follow-up.

Tertiary outcomes at the latest follow-up included post-operative complications (dislocation, aseptic loosening, periprosthetic fracture, infection, and pain), implant-related events, non-implant-related events, hospitalisation outcomes, and clinical scores (Harris Hip Score (HHS), and Merle D'Aubigne and Postel score (MDP)).

We evaluated the methodology of the chosen studies using the Risk Of Bias In Non-randomised Studies of Interventions (ROBINS-I) tool.^29^ The tool is appropriate in this context given its relevance to non-randomised intervention studies and ability to provide a structured approach to discerning reliability and validity. It encompasses seven constituent domains: confounding bias, selection bias, classification bias, intervention bias, missing data bias, measurement bias, and reporting bias. Responses to these domains were categorised as “yes”, “probable yes”, “probable no”, “no”, or “unavailable”. Overall bias was then graded as “low”, “moderate”, “serious”, or “critical”. Two reviewers (SS, FJR) independently assessed the methodological quality, with arbitration provided by a senior author (AP).

The primary and secondary outcomes were inspected for publication bias and small-study effects using funnel plots. A regression-based Egger test was also conducted.^30^ Statistical significance was set as *p* < 0.05.^31^

In the synthesis, odds ratios (ORs) or pooled proportions were used for dichotomous outcomes, and mean or median for continuous variables.

Initially, a narrative synthesis summarised the study characteristics, study population demographics, and outcomes. Values were presented as reported in the original studies without rounding to account for variable significant figure reporting across studies, and to ensure the accuracy and integrity of the data, Statistical analysis was conducted using StataMP (version 17.0, StataCorp, USA). As the primary and secondary outcomes were dichotomous variables, a meta-analysis was performed using a random-effects maximum-likelihood (REML) logistic regression model via the metan package.

To estimate the between-study heterogeneity in the REML model, the DerSimonian-Laird estimate of Tau^2^ was used. The inverse-variance method was used to calculate the weights assigned to each study in the meta-analysis. The summary effect confidence intervals (CIs) were calculated using the Wald-type CI method.^32^

Forest plots were constructed for the primary and secondary outcomes at the latest reported follow-up, and pooled estimates with 95 % CIs were synthesised using the metaprop package.^33^

Forest plots were examined to ascertain the level of interstudy heterogeneity, and Chi^2^ and *I*^2^ statistical methods were employed.^34^ The *I*^2^ statistic was interpreted according to the Cochrane Handbook for Systematic Reviews of Interventions.^32^

## 3 Results

**Fig. 2:**
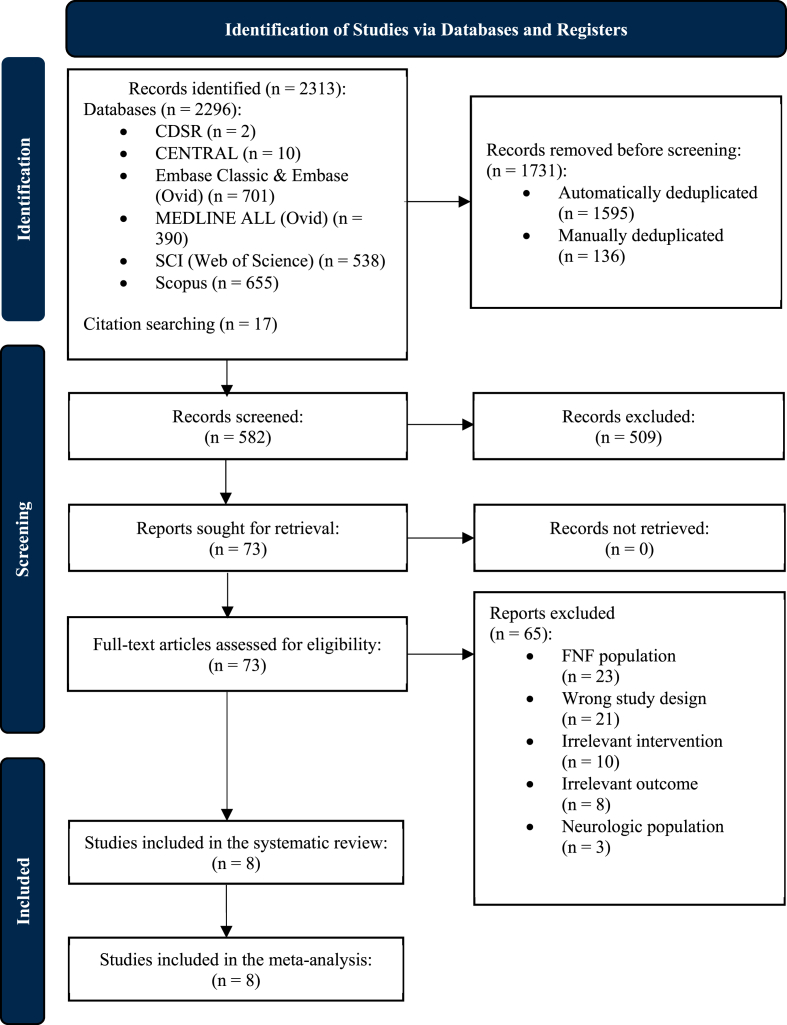
A PRISMA flow diagram, providing a visual representation of the systematic review process, including the study identification, screening, and inclusion phases, and the consequent number of included studies in the systematic review and meta-analysis. n: number.

2313 records were identified. After deduplication, title and abstract screening and full-text review, eight comparative, non-randomised studies remained for inclusion in the qualitative and quantitative synthesis^35^, ^36^, ^37^, ^38^, ^39^, ^40^, ^41^, ^42^ (Fig. 2).

Two involved pTHAs^35^^,^^36^ and six involved rTHAs.^37^, ^38^, ^39^, ^40^, ^41^, ^42^ All included studies were published in the last eight years and involved implants inserted between 1995 and 2019 (Table 2).

**Table 2:** **Included Study Characteristics.***ROS: Retrospective Observational Study; CCS: Case-Controlled Study; POS: Prospective Observational Study; LAR: Local Arthroplasty Register; ID: Institutional Database; IJR: International Joint Registry; ALTR: Adverse Local Tissue Reaction; OA: Osteoarthritis; DL: Direct Lateral; PLA: Posterolateral Approach; LDH: Large Diameter Head; n: number; NR: Not Reported;*^†^: (Stryker Orthopaedics, USA); *: (Zimmer Biomet, USA);^◆^*: (Amplitude, France);*^∫^*: (Smith & Nephew, United Kingdom);*^‡^*: (Groupe Lépine, France);*^§^*: (Ceraver Osteal, France).*

Study	Country	Level of Evidence	Study Design	Intervention Period	Data Source	Indication for Surgery	Surgical Approach	Cement Use	DM Group Prosthesis Manufacturer	FB Group Prosthesis Manufacturer	Total Hips, n	DM Group Hips, n	FB Group Hips, n
**Studies Reporting Primary THAs**													
Epinette et al. 2015^35^	France	III	POS	2007–2011	ID	All Causes	PLA	Uncemented	ADM Group^†^	Trident PSL^†^	273	143	130
Kreipke et al. 2019^36^	Denmark	III	POS	1995–2013	IJR	OA	Multiple	Multiple	Avantage*; Saturn^◆^; POLARCUP^∫^	Various	4554	2277	2277
**Studies Reporting Revision THAs**													
Brüggemann et al. 2018^37^	Sweden	III	ROS	2008–2016	LAR	All Causes	Multiple	Multiple	Avantage* cemented into a larger TM Modular, Trilogy TM or TM Revision shell*	Various	184	69	115
Di Martino et al. 2023^38^	Italy	III	ROS	2000–2019	LAR	All Causes	NR	Multiple	Various	Unspecified. Sizes:≤28 mm; 32 mm; ≥36 mm	253	57	196
Harwin et al. 2018^39^	USA	III	ROS	2011–2015	ID	All Causes	DL	Uncemented	X3 Modular DM Acetabular System^†^	Trident Acetabular System^†^	255	85	170
Hernigou et al. 2016^40^	France	IV	CCS	2005–2010	ID	Cup Loosening	PLA	Cemented	Quattro^‡^;Ceraver DM Group device^§^	32-mm^§^	67	35	32
Klemt et al. 2020^41^	USA	III	ROS	NR	ID	ALTR	PLA	NR	Various	Unspecified. Sizes: Non-LDH (diameter ≤32 mm); LDH (diameter >32 mm)	210	42	168
Spaans et al. 2018^42^	The Netherlands	III	ROS	2007–2013	ID	All Causes	PLA	Cemented	Avantage cup*	Various	202	102	100

Overall, there were 2810 DM implants, consisting of 2420 pTHAs and 390 rTHAs. There were 3188 FB implants consisting of 2407 pTHAs and 781 rTHAs.

Both pTHA studies^35^^,^^36^ were level III prospective observational studies from Europe. One studied institutional database data,^35^ and the other used international joint registry data.^36^ The two pTHA studies provided 2420 DM implants versus 2407 FB implants. One study reported a mean overall follow-up of four years (range two to six),^35^ with no specific details regarding the different cohorts. The other study reported a median overall follow-up of three years (range one to five),^36^ with a median of three years (range of one to five) for both of its cohorts.

Five rTHA studies were level III retrospective observational studies,^37^, ^38^, ^39^^,^^41^^,^^42^ and one was a level IV case-controlled study.^40^ Four studies were from Europe,^37^^,^^38^^,^^40^^,^^42^ and two were from North America.^39^^,^^41^ Four studied institutional database data,^39^, ^40^, ^41^, ^42^ and two used local arthroplasty registry data.^37^^,^^38^ These six studies provided 390 DM implants versus 781 FB implants. The mean overall follow-up was 8 years, with a mean of seven years for the DM cohort and nine years for the FB cohort.

**Fig. 3:**
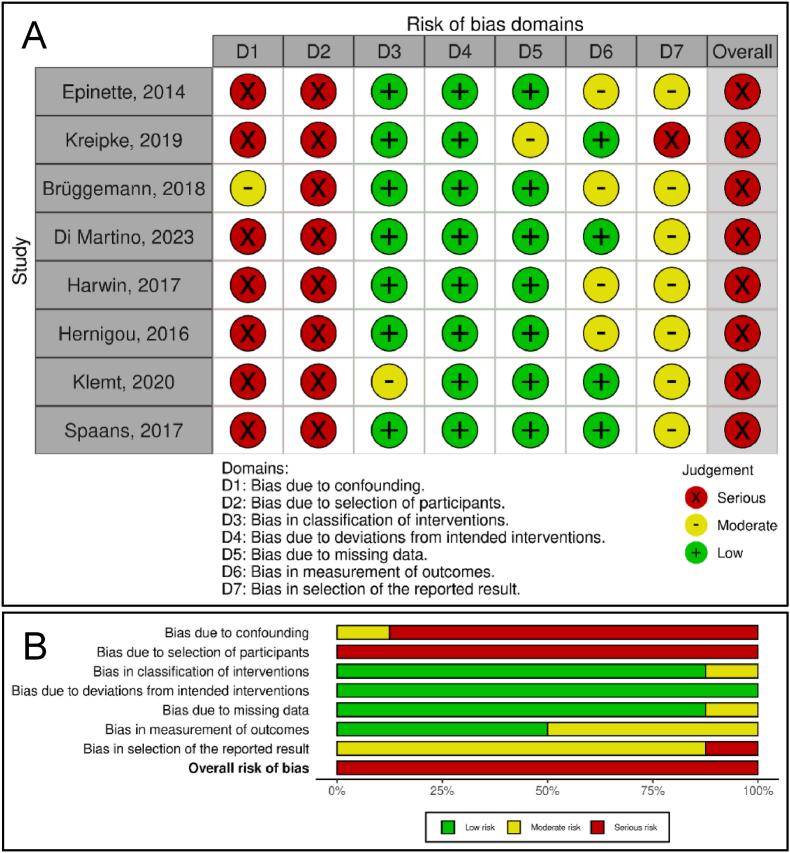
(A) A traffic light plot of the domain-level risk-of-bias judgement for each included study. The colours indicate the deemed risk of bias, with green indicating low risk, yellow indicating moderate risk and red indicating serious risk. The overall risk of bias corresponds to the worst judgement obtained across all of a study's domains. (B) Unweighted bar plots illustrating the distribution of the risk of bias judgements within each domain. The risk-of-bias assessment involved careful examination of each study and comparison against an idealised study design to evaluate the risk of bias in each domain. This required in-depth methodological and content expertise to identify potential biases and assess their impact on the overall results. The idealised study design was a hypothetical benchmark that allowed the assessment of each included study in a consistent and rigorous fashion.

**Fig. 4:**
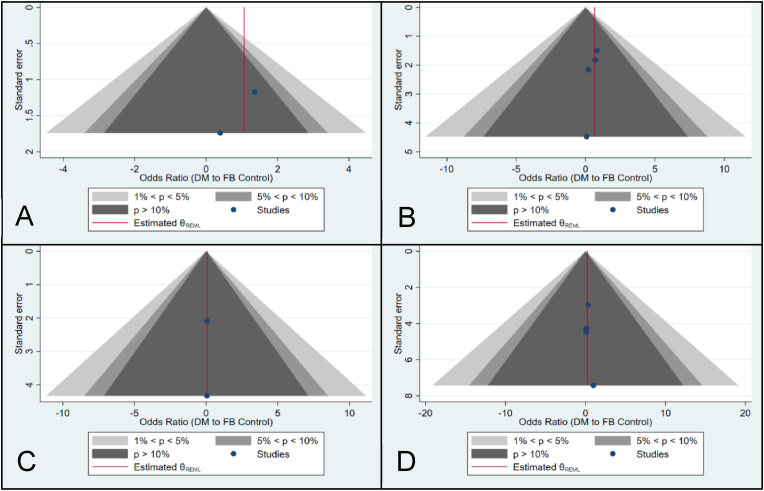
Contour-enhanced funnel plots, shown as the distribution precision (Standard Error, y-axis) against the effect size estimate (Odds Ratio, x-axis). Milestones of statistical significance indicated at p = 0.01, 0.05 and 0.10 (A) (Upper Left) All-cause revision in the pTHA studies. (B) (Upper Right) All-cause revision in the rTHA studies. (C) (Lower Left) Revision due to dislocation in the pTHA studies. (D) (Lower Right) Revision due to dislocation in the rTHA studies.

The ROBINS-I tool results were visualised as a traffic light plot and summary plot using the Robvis tool^43^ (Fig. 3, Supplementary File 2). All included studies had “serious” overall bias, primarily due to issues regarding confounding and participant selection. However, as no examined studies had a “critical” overall bias none were excluded. The funnel plots (Fig. 4), and Egger test did not indicate evidence of publication bias.

The included studies’ patient demographics are presented in Table 3.

**Table 3:** **Patient Demographics.** BMI: Body Mass Index; SD: Standard Deviation; n: number; NR: Not Reported. Merged cells indicate overall values for the total study population and not for individual study cohorts.

Study	Total Hips, n	DM Group Hips, n	FB Group Hips, n	Mean Overall FU (Range)	DM Group Mean FU (Range)	FB Group Mean FU (Range)	DM Group Mean Age in years ± SD (Range)	FB Group Mean Age in years ± SD (Range)	DM Group Females (%)	FB Group Females (%)	DM Group Mean BMI kg/m^2^ ± SD (Range)	FB Group Mean BMI kg/m^2^ ± SD (Range)
**Studies Reporting Primary THAs**												
Epinette et al. 2015^35^	273	143	130	4.13 (2–6)	NR	NR	70.63 ± 9.498 (41–95)	65.50 ± 12.724 (27–94)	64.0 %	57.6 %	28.448 ± 5.267 (17.70–44.44)	28.425 ± 5.436 (18.10–48.80)
Kreipke et al. 2019^36^	4554	2277	2277	*MEDIAN: 3.01 (1.31 - 5.40)*	*MEDIAN: 2.99 (1.31-5.40)*	*MEDIAN: 3.20 (1.47-5.39)*	75 ± NR (NR)	76 ± NR (NR)	60.6 %	59.9 %	NR	NR
**Studies Reporting Revision THAs**												
Brüggemann et al. 2018^37^	184	69	115	4.9 (0.5–8.9)	NR	NR	67 ± NR (35–88)		50.7 %	51.3 %	NR	NR
Di Martino et al. 2023^38^	253	57	196	10	10	10	74.8 ± NR (48–92)	71.2 ± NR (33–96)	65.6 %		NR	NR
Harwin et al. 2018^39^	255	85	170	8 (2–14)	4 (2–6)	10 (6–14)	67 ± NR (42–90)	69 ± NR (42–88)	49.4 %	47.1 %	31 ± NR (20–49)	32 ± NR (21–46)
Hernigou et al. 2016^40^	67	35	32	7 (5–10)	7 (5–10)	7 (5–10)	74.7 ± 6.4 (NR)	72.1 ± 6.4 (NR)	57.1 %	43.8 %	NR	NR
Klemt et al. 2020^41^	210	42	168	4 (2.8–8.6)	NR	NR	55 ± 8 (NR)	61 ± 13 (NR)	59.5 %	54.8 %	28 ± 8 (NR)	26 ± 7 (NR)
Spaans et al. 2018^42^	202	102	100	2.3 (0.25–7)	NR	NR	73.1 ± 8.5 (NR)	65.4 ± 12.4 (NR)	60.8 %	67.0 %	26.3 ± 4.5 (NR)	27.2 ± 5.7 (NR)

The mean age of all included pTHA patients was 72 years (range 66–76). The mean age of DM pTHA patients was 73 (range 71–75) and 71 (range 66–76) for FB pTHA patients. There were 1472 (61 %) and 1439 (60 %) female DM pTHA and FB pTHA patients, respectively.

The mean age of all included rTHA patients was 68 years (range 55–75). The mean age of DM rTHA patients was 69 years (range 55–75) and 68 years (range 61–72) for FB rTHA patients. There were 221 (57 %) and 441 (56 %) female DM rTHA and FB rTHA patients, respectively.

The primary and secondary outcome data are presented in Table 4. Note that one study did not report the primary or secondary outcomes but was still included in the narrative synthesis.^41^

**Table 4:** **All-Cause Revisions and Revisions due to Dislocation.** FU: Follow-Up; SD: Standard Deviation; n: number; NR: Not Reported.

Study	Total Hips, n	DM Group Hips, n	FB Group Hips, n	Mean Overall FU (Range)	DM Group Mean FU (Range)	FB Group Mean FU (Range)	DM Group Revisions (All Cause), n	FB Group Revisions (All Cause), n	DM Group Revisions (due to dislocation), n	FB Group Revisions (due to dislocation), n
**Studies Reporting Primary THAs**										
Epinette et al. 2015^35^	273	143	130	4.13 (2–6)	NR	NR	5	11	0	7
Kreipke et al. 2019^36^	4554	2277	2277	*MEDIAN: 3.01 (1.31 - 5.40)*	*MEDIAN: 2.99 (1.31-5.40)*	*MEDIAN: 3.20 (1.47-5.39)*	97	72	2	24
**Studies Reporting Revision THAs**										
Brüggemann et al. 2018^37^	184	69	115	4.9 (0.5–8.9)	NR	NR	2	15	0	11
Di Martino et al. 2023^38^	253	57	196	10 (NR)	10 (NR)	10 (NR)	9	36	0	11
Harwin et al. 2018^39^	255	85	170	8 (2–14)	4 (2–6)	10 (6–14)	4	11	1	6
Hernigou et al. 2016^40^	67	35	32	7 (5–10)	7 (5–10)	7 (5–10)	0	5	0	5
Klemt et al. 2020^41^	210	42	168	4 (2.8–8.6)	NR	NR	NR	NR	NR	NR
Spaans et al. 2018^42^	202	102	100	2.3 (0.25–7)	NR	NR	5	7	0	0

Although data were collected on several tertiary outcomes, data were insufficient in certain domains for meaningful inter-study comparisons. Only domains with sufficient data were included in the narrative and graphical synthesis (Table 5, Table 6).

1. Primary Outcome: All-Cause Revision

**Table 5:** **Other Clinical Outcomes.** n: number; NR: Not Reported.

Study	Total Hips, n	DM Group Hips, n	FB Group Hips, n	DM Group Dislocations, n	FB Group Dislocations, n	DM Group Aseptic Loosenings, n	FB Group Aseptic Loosenings, n	DM Group Periprosthetic Fractures, n	FB Group Periprosthetic Fractures, n	DM Group Infections, n	FB Group Infections, n
**Studies Reporting Primary THAs**											
Epinette et al. 2015^35^	273	143	130	0	7	0	0	3	1	2	3
Kreipke et al. 2019^36^	4554	2277	2277	NR	NR	NR	NR	NR	NR	NR	NR
**Studies Reporting Revision THAs**											
Brüggemann et al. 2018^37^	184	69	115	1	14	2	2	0	0	0	1
Di Martino et al. 2023^38^	253	57	196	0	11	4	11	2	1	1	4
Harwin et al. 2018^39^	255	85	170	1	6	1	3	1	2	1	2
Hernigou et al. 2016^40^	67	35	32	1	7	0	0	0	0	0	0
Klemt et al. 2020^41^	210	42	168	0	26	NR	NR	1	9	0	7
Spaans et al. 2018^42^	202	102	100	3	8	1	1	NR	NR	4	1

**Table 6:** **Functional Scores at the Latest Follow-Up.** HHS: Harris Hip Score; MDP: Merle D'Aubigne and Postel score; SD: Standard Deviation; NR: Not Reported. Merged cells indicate overall values for the total study population and not for individual study cohorts.

Study	DM Group Mean HHS ± SD (Range)	FB Group Mean HHS ± SD (Range)	DM Group Mean MDP Score ± SD (Range)	FB Group Mean MDP Score ± SD (Range)
**Studies Reporting Primary THAs**				
Epinette et al. 2015^35^	97.42 ± 4.098 (NR)	98.07 ± 3.530 (NR)	17.42 ± 0.966 (NR)	17.56 ± 0.817 (NR)
Kreipke et al. 2019^36^	NR	NR	NR	NR
**Studies Reporting Revision THAs**				
Brüggemann et al. 2018^37^	77 (25–100)		NR	NR
Di Martino et al. 2023^38^	NR	NR	NR	NR
Harwin et al. 2018^39^	88 ± NR (44–100)	86 ± NR (48–100)	NR	NR
Hernigou et al. 2016^40^	NR	NR	14.32 ± 1.72 (12–17)	14.24 ± 1.84 (12–16)
Klemt et al. 2020^41^	NR	NR	NR	NR
Spaans et al. 2018^42^	NR	NR	NR	NR

**Fig. 5:**
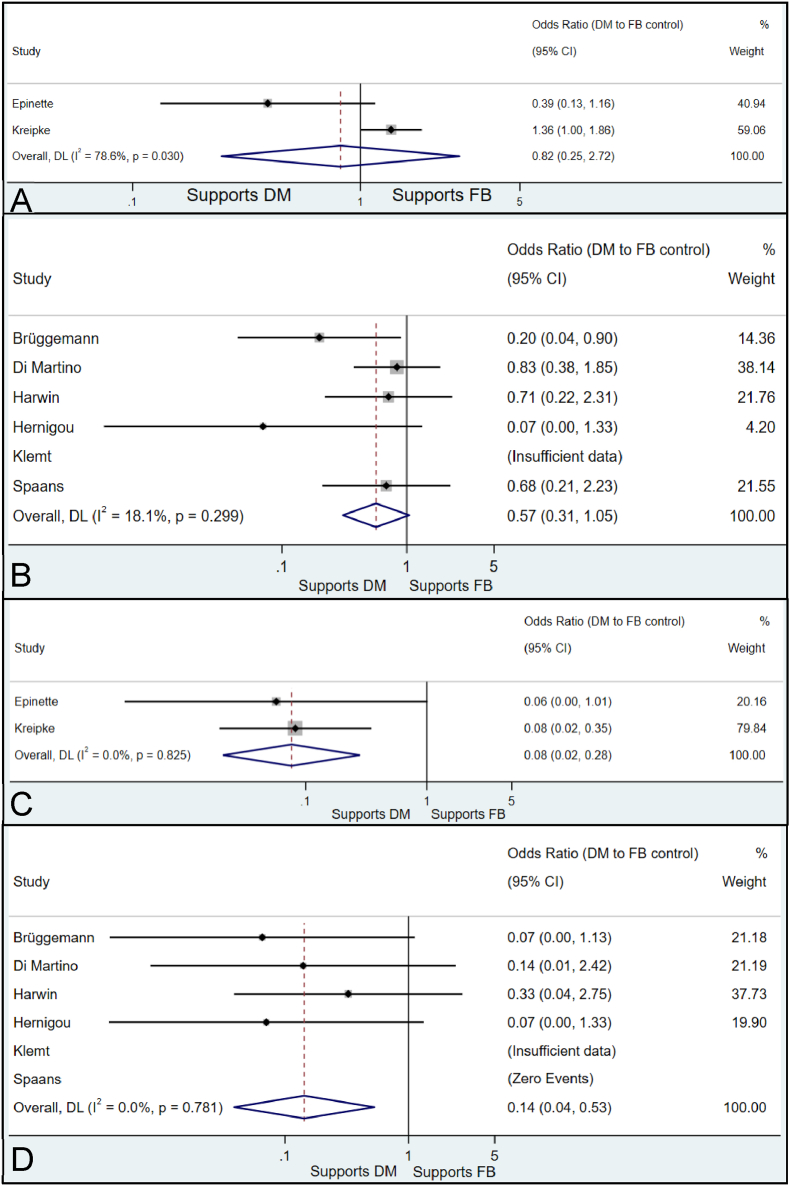
Forest plots. Shown as the individual studies (y-axis) against the effect size estimate (Odds Ratio, x-axis). The effect size estimate is portrayed by a point estimate and 95 % CIs (horizontal line) that reflect the precision of the estimate. The null effect line is portrayed as a black vertical line intersecting one. The overall weighted effect size is given as a dashed vertical red line and diamond. DL: DerSimonian-Laird. (A) All-cause revision in the pTHA studies. (B) All-cause revision in the rTHA studies. (C) Revision due to dislocation in the pTHA studies. (D) Revision due to dislocation in the rTHA studies.

In the pTHA studies the pooled OR in the DM cohort versus the FB cohort was 0.82 (95 % CI 0.25–2.72), indicating a tendency towards lower revision rates in the DM cohort, although the difference was statistically insignificant due to the intersection of the null effect line. The *I*^2^ statistic of 78.6 % (*p* = 0.030) indicated substantial and statistically significant heterogeneity (Fig. 5a).

In the rTHA studies the pooled OR in the DM cohort versus the FB cohort was 0.57 (95 % CI 0.31–1.05), indicating a tendency towards lower revision rates in the DM cohort, although the difference was statistically insignificant due to the intersection of the null effect line. The *I*^2^ statistic of 18.1 % (*p* = 0.299) indicated low and statistically insignificant heterogeneity (Fig. 5b).

2. Secondary Outcome: Revision due to Dislocation

In the pTHA studies the pooled OR in the DM cohort versus the FB cohort was 0.08 (95 % CI 0.02–0.28), indicating a statistically significant benefit favouring DM. The risk of dislocation was 12.5 times greater in FB implants vs DM. The *I*^2^ statistic of 0.0 % (*p* = 0.825) indicated an absence of heterogeneity amongst the included studies, but note that whilst the effect sizes are similar, it cannot be known with certainty that heterogeneity was absent (Fig. 5c).

In the rTHA studies the pooled OR in the DM cohort versus the FB cohort was 0.14 (95 % CI 0.04–0.53), favouring DM with statistical significance. Therefore, FB implants were 7.1 times more likely to dislocate when compared with DM. The *I*^2^ statistic of 0.0 % (*p* = 0.781) indicated a similar absence of heterogeneity amongst the included studies, portraying consistent effect sizes (Fig. 5d).

3. Tertiary Outcomes: Post-operative Complications & Clinical Scores

Kreipke et al.^36^ reported dislocation, aseptic loosening, infection, and periprosthetic fracture (PPF), as causes of revision in its cohorts, but did not report the standalone post-operative complication data specified as this study's tertiary outcomes, hence, pooled proportions could not be obtained for the pTHA studies overall. The following observations were made within the Epinette et al. study,^35^ amongst 143 DM implants and 130 FB implants, no dislocations occurred in the DM cohort, in contrast to an incidence of 5.4 % in the FB cohort. No aseptic loosening was observed in either cohort, and there was an increased incidence of PPFs in the DM cohort compared to the FB cohort, with 2.1 % and 0.8 %, respectively. Infection was decreased in DM implants compared to FB implants, with 1.4 % and 2.3 % incidence, respectively, although this was statistically insignificant.

In the rTHA studies,^37^, ^38^, ^39^, ^40^, ^41^, ^42^ the pooled proportions for dislocation^37^, ^38^, ^39^, ^40^, ^41^, ^42^ were significantly lower for DM implants at 1 %, compared to FB (10 %). The incidence of aseptic loosening^37^, ^38^, ^39^, ^40^^,^^42^ was similar between DM and FB implants, with both having an incidence of 2 %. The pooled proportions for infection^37^, ^38^, ^39^, ^40^, ^41^, ^42^ favoured DM over FB implants, with DM at 1 % and FB at 2 %. Finally, the incidence of PPF^37^, ^38^, ^39^, ^40^, ^41^ was similar for both DM and FB implants, with both cohorts having an incidence of 1 %.

Clinical scores including the HHS, and MDP, were reported in two,^37^^,^^39^ and one^40^ rTHA study, respectively (Table 6). Brüggemann et al.^37^ only reported the combined HHS for the DM and FB cohort, however, so studies could not be compared using either score.

## 4 Discussion

Although previous studies have suggested potential benefits in the use of the DM design in high-risk patient populations,^23^, ^24^, ^25^, ^26^ there is limited strong evidence that determines whether the use of DM or FB implants in pTHA and rTHA leads to different outcomes in terms of all-cause revision, revision due to dislocation, and other complications.

Our results suggest that DM implants tend to confer lower all-cause revision surgery rates than FB implants in pTHA and rTHA, but this could not be proven statistically due to inter-study heterogeneity. Nevertheless, recent literature supports the finding of reduced all-cause revision in DM designs.^44^^,^^45^

Regarding revision due to dislocation, DM implants exhibited a clear benefit over FB implants in both pTHA and rTHA studies. The I^2^ statistic of 0.0 % (p = 0.825) indicated consistent effect sizes in both the primary and revision settings, but it was not concluded definitively that there was no statistical heterogeneity between studies. The literature supports this benefit, however.^44^

DM implants showed a significant reduction in implant dislocation, especially in the revision context. This aligns with the theoretical advantages of DM implants, such as increased ROM and reduced risk of impingement, which are particularly beneficial in revision cases that inherently feature altered anatomy.

Intraprosthetic dislocation (IPD), a unique complication of the DM design, has raised scepticism amongst some operators. However, reassuringly, the absence of IPDs at mid-term follow-up lends support to the assertion that they are rare occurrences in contemporary DM designs.^26^^,^^46^

The data suggested that DM implants had a lower incidence of infection compared to FB implants in both pTHA and rTHA studies. There was a higher incidence of PPFs in the DM cohort compared to the FB cohort in one pTHA study,^35^ and the incidence of aseptic loosening was similar between cohorts in both pTHA and rTHA studies. Functional scores could not be compared between studies due to limited reporting, restricting our ability to draw definitive conclusions.

The International Society of Arthroplasty Registries (ISAR) recommends all-cause surgical revision as the primary outcome measure for benchmarking,^47^ as implant survival data can result in biased conclusions.^48^

The study's pre-defined protocol was published *a priori* and is accessible via PROSPERO, ensuring a fully transparent methodology.

A potential limitation to consider is publication bias, where studies with positive or statistically significant results are more likely to be published. Despite thorough database searches and efforts to contact authors, there remains a possibility that studies without significant findings were missed. Whilst the funnel plots appear symmetrical upon visual inspection and the Egger's test indicated no statistically significant publication bias, its power and sensitivity were limited by the small number of included studies, and should be interpreted with care.

The ROBINS-I tool reflected a “serious” risk of bias in all studies, mainly due to issues in confounding and participant selection. Other potential concerns include attrition bias due to the differential loss of participants between the intervention and control groups, validation bias due to non-standardised methods of component assessment between studies, and verification bias as the outcome assessors were not blinded to treatment allocation. However, the results were reminiscent of Donovan et al.‘s findings,^49^ who assessed a similar distribution of bias amongst their included studies.

Seven of the studies were level III evidence^35^, ^36^, ^37^, ^38^, ^39^^,^^41^^,^^42^ and one was level IV,^40^ hence, the study results may not be as strong as higher-level evidence, such as randomised-controlled trials (RCTs). The abundance of retrospective studies with a lower level of evidence is a recognised flaw in the orthopaedic literature,^50^ but data pooling improves statistical power to provide more robust evidence for clinical decision-making. Conducting sensitivity analyses or a network meta-analysis once enough studies are available in the literature would be valuable to further explore the effects of different levels of evidence.

The included patients had various aetiologies, including primary osteoarthritis,^36^ cup loosening,^40^ and adverse local tissue reaction.^41^ This may have caused an overestimation of dislocation incidence in the FB cohort compared to the literature.^51^ Moreover, surgical factors varied, including approach, cement use, and the number of operating surgeons. The outer femoral head diameter varied between DM implant designs, with larger femoral heads potentially giving additional stability to cause confounding. Such heterogeneity poses a potential limitation. More homogeneous patient populations and uniform surgical techniques would improve external validity in future reviews for definitive conclusions on the comparative effectiveness of DM and FB implants in THA.

Analysis at the latest follow-up was appropriate given the eligible studies' variable follow-up lengths. Other systematic reviews have recognised the literature's propensity for short-term follow-up.^44^^,^^52^, ^53^, ^54^, ^55^ The rTHA cohort had longer follow-up than the included pTHA studies, allowing for the detection of later complications and failures that may not have manifested in the short term. However, the long-term impacts of DM implants remain uncertain. Previous studies have raised concerns about metallosis^56^^,^^57^ and wear of the polyethylene liner.^58^ It is therefore important that surgeons and future studies monitor for these potential complications.

Despite the included studies' longer follow-up time, only four studies (one pTHA, three rTHA) reported clinical outcomes, limiting this study's ability to investigate an integral aspect of the lived patient experience.^59^ Although the results were favourable, the use of different score measurements (HHS or MDP) prevented an adequate comparison analysis. Various other factors that were originally planned for analysis were reported inconsistently amongst the included studies. Future studies should be aware of this feature when planning their included variables to streamline the data extraction process.

Only one small study reported the tertiary outcomes in pTHA,^35^ the other national registry study could not report on these items.^36^

No well-powered RCTs have explored the use of DM implants to mitigate post-operative dislocation risk after THA. This is due to the high cost, large sample sizes and long-term follow-up required for joint replacement study.^48^^,^^60^ However, two proposed national registry-based study designs have been described in the literature.^61^^,^^62^

The current study's findings have important clinical implications. DM implants may reduce the risk of revision surgery due to dislocation compared to FB implants in both pTHA and rTHA patients. This information can be used to inform clinical decision-making regarding implant selection for patients, for instance, favouring the DM design in patients with an elevated risk of dislocation, such as patients with spinal disease, or revision surgery for recurrent dislocation.

## 5 Conclusion

This study compared the outcomes of DM and FB implants in the primary and revision settings. The results suggest that DM implants tend towards lower all-cause revision surgery rates than FB implants in both contexts. A clear benefit favouring DM over FB implants was observed in both pTHA and rTHA studies in revision due to dislocation. The study provided valuable insights regarding the incidence of various post-operative complications between DM and FB implants.

Our findings suggest that DM implants may have some advantages over FB implants for THA in the primary and especially revision settings. Further prospective studies are warranted to validate these findings and to explore other outcomes of interest.

## Author contributions

All authors contributed to the study conceptualisation and design. Material preparation, data collection and analysis were performed by Sarup Saroha and Firas J Raheman. The first draft of the manuscript was written by Sarup Saroha and all authors commented on previous versions of the manuscript. All authors read and approved the final manuscript.
