# Supplementary material for: Dual-mobility implants in primary and revision total hip arthroplasty: A systematic review and meta-analysis
Source: J Clin Orthop Trauma. 2024 Jul 18;54:102495. doi: 10.1016/j.jcot.2024.102495 (PMC11324850; doi:10.1016/j.jcot.2024.102495)
Supplement: Multimedia component 1 [file mmc1.docx]

**ROBINS-I Tool Signalling Questions.**

*POS: Prospective Observational Study; CCS: Case-Controlled Study; ROS: Retrospective Observational Study; Y: Yes; PY: Probable Yes; N: No; PN: Probable No; NA: Not Applicable; NI: No Information.*
