# Supplementary material for: Dual-mobility implants in primary and revision total hip arthroplasty: A systematic review and meta-analysis
Source: J Clin Orthop Trauma. 2024 Jul 18;54:102495. doi: 10.1016/j.jcot.2024.102495 (PMC11324850; doi:10.1016/j.jcot.2024.102495)
Supplement: Multimedia component 2 [file mmc2.docx]

## CDSR and CENTRAL Search Strategy (The Cochrane Library)

| Line | Query | CDSR | CENTRAL |
| --- | --- | --- | --- |
| 1 | MeSH descriptor: [Hip] explode all trees | 2 | 485 |
| 2 | MeSH descriptor: [Hip Joint] explode all trees | 4 | 1,139 |
| 3 | MeSH descriptor: [Femur Head] explode all trees | 1 | 125 |
| 4 | MeSH descriptor: [Acetabulum] explode all trees | 0 | 232 |
| 5 | MeSH descriptor: [Pelvis] explode all trees | 35 | 1,310 |
| 6 | MeSH descriptor: [Hip Injuries] explode all trees | 38 | 2,256 |
| 7 | MeSH descriptor: [Wounds and Injuries] explode all trees | 441 | 32,740 |
| 8 | #1 or #2 or #3 or #4 or #5 or #6 or #7 | 477 | 35,526 |
| 9 | MeSH descriptor: [Arthroplasty, Replacement, Hip] explode all trees | 17 | 2,346 |
| 10 | MeSH descriptor: [Hip Prosthesis] explode all trees | 2 | 1,238 |
| 11 | (replac* OR arthropl* OR surg* OR prosth* OR interv*):ti,ab,kw | 7,873 | 863,847 |
| 12 | #9 or #10 or #11 | 7,873 | 863,847 |
| 13 | ("dual-mobility" or "dual mobility" or "tripolar" or "tri-polar" or "double-mobility" or "double mobility" or "mobile-bearing" or "mobile bearing" or "dual construct" or "dual-construct"):ti,ab,kw | 3 | 271 |
| 14 | (primar* or revis*):ti,ab,kw | 8,524 | 532,951 |
| 15 | #8 and #12 and #13 and #14 | 2 | 10 |

## Embase and MEDLINE Search Strategy (Ovid).

| Line | Query | Embase | MEDLINE |
| --- | --- | --- | --- |
| 1 | exp "HIP"/ or exp "HIP JOINT"/ or exp "FEMORAL HEAD"/ or exp "ACETABULUM"/ or exp "PELVIS"/ or exp "hip injuries"/ or exp "injuries"/ | 3,041,834 | 1,071,789 |
| 2 | exp "ARTHROPLASTY, REPLACEMENT, HIP"/ or exp "HIP PROSTHESIS"/ | 59,871 | 47,037 |
| 3 | (replac* or arthrop* or surg* or prosth* or interv*).ti,ab. | 6,929,942 | 4,961,996 |
| 4 | 2 or 3 | 6,937,284 | 4,967,170 |
| 5 | ("dual-mobility" or "dual mobility" or "tri-polar" or "tripolar" or "double-mobility" or "double mobility" or "mobile-bearing" or "mobile bearing" or "dual-construct" or "dual construct").ti,ab. | 3,080 | 2,550 |
| 6 | (primar* or revis*).ti,ab. | 3,403,905 | 2,358,712 |
| 7 | 1 and 4 and 5 and 6 | 704 | 390 |

## Scopus Search Strategy Returning 655 Results.

( ( ( TITLE-ABS-KEY ( "HIP" ) OR TITLE-ABS-KEY ( "HIP JOINT" ) OR TITLE-ABS-KEY ( "FEMORAL HEAD" ) OR TITLE-ABS-KEY ( "ACETABULUM" ) OR TITLE-ABS-KEY ( "PELVIS" ) OR TITLE-ABS-KEY ( "hip injuries" ) OR TITLE-ABS-KEY ( "injuries" ) ) ) ) AND ( ( ( ( TITLE-ABS-KEY ( "ARTHROPLASTY, REPLACEMENT, HIP" ) OR TITLE-ABS-KEY ( "HIP PROSTHESIS" ) ) ) ) OR ( ( ( TITLE-ABS-KEY ( "replac*" ) OR TITLE-ABS-KEY ( "arthrop*" ) OR TITLE-ABS-KEY ( "surg*" ) OR TITLE-ABS-KEY ( "prosth*" ) OR TITLE-ABS-KEY ( "interv*" ) ) ) ) ) AND ( ( ( TITLE-ABS-KEY ( "dual-mobility" ) OR TITLE-ABS-KEY ( "dual mobility" ) OR TITLE-ABS-KEY ( "tri-polar" ) OR TITLE-ABS-KEY ( "tripolar" ) OR TITLE-ABS-KEY ( "double-mobility" ) OR TITLE-ABS-KEY ( "double mobility" ) OR TITLE-ABS-KEY ( "mobile-bearing" ) OR TITLE-ABS-KEY ( "mobile bearing" ) OR TITLE-ABS-KEY ( "dual-construct" ) OR TITLE-ABS-KEY ( "dual construct" ) ) ) ) AND ( ( ( TITLE-ABS-KEY ( "primar*" ) OR TITLE-ABS-KEY ( "revis*" ) ) ) )

## Web of Science (Science Citation Index) Search Strategy Returning 538 Results.

((HIP OR "HIP JOINT" OR "FEMORAL HEAD" OR ACETABULUM OR PELVIS OR "hip injuries" OR injuries) AND (("ARTHROPLASTY, REPLACEMENT, HIP" OR "HIP PROSTHESIS") OR (replac* OR arthrop* OR surg* OR prosth* OR interv*)) AND ("dual-mobility" OR "tri-polar" OR "double-mobility" OR "mobile-bearing" OR "dual-construct" OR "dual mobility" OR "tripolar" OR "double mobility" OR "mobile bearing" OR "dual construct") AND (primar* OR revis*))
